# Broccoli, Artichoke, Carob and Apple By-Products as a Source of Soluble Fiber: How It Can Be Affected by Enzymatic Treatment with Pectinex^®^ Ultra SP-L, Viscozyme^®^ L and Celluclast^®^ 1.5 L

**DOI:** 10.3390/foods14010010

**Published:** 2024-12-25

**Authors:** Pablo Ayuso, Rocío Peñalver, Jhazmin Quizhpe, María de los Ángeles Rosell, Gema Nieto

**Affiliations:** Department of Food Technology, Nutrition and Food Science, Veterinary Faculty, University of Murcia, Regional Campus of International Excellence “Campus Mare Nostrum”, 30100 Murcia, Spain; pablo.ayuson@um.es (P.A.); rocio.penalver@um.es (R.P.); jhazminedith.quizhper@um.es (J.Q.); mariaangeles.rosellp@um.es (M.d.l.Á.R.)

**Keywords:** prebiotic, soluble fiber, natural extract, food additives, enzymatic treatment

## Abstract

Dietary fiber (DF), and especially soluble dietary fiber (SDF), is a nutrient of particular interest today because of its anti-inflammatory role and its ability to reduce cardiovascular risk. Therefore, the enhancement of SDF in foods using different techniques has become a promising field of research. In order to prove the possibility of increasing this SDF content, the effects of different commercial enzymes (Pectinex^®^ Ultra SP-L, Viscozyme^®^ L and Celluclast^®^ 1.5 L) were tested on a variety of carob (CE), artichoke (ARE), apple (APE) and broccoli (BE) by-product extracts. Enzymatic treatment significantly affected SDF content in all by-products, showing the greatest increases for CE, ARE and APE using Celluclast^®^ 1.5 L, while Viscozyme^®^ L obtained the best results after application in BE. On the other hand, positive results were reported in the solubility, WHC and FAC of the by-products due to the enzymatic treatment, being increased in all extracts analyzed. Moreover, a general increase in antioxidant capacity (FRAP, ABTS and DPPH) was observed after enzymatic treatment. Finally, high yields were obtained after the application of the enzymatic processes, reaching values of 80–85% for each food by-product. These results evidenced a potential revalorization of carob, artichoke, apple and broccoli by-products after enzymatic treatment, improving its nutritional and physicochemical properties, revealing a possible application as a higher value-added ingredient.

## 1. Introduction

Consumers are increasingly looking for products with a high content of dietary fiber (DF), which is becoming a key nutrient in the human diet. Dietary fiber is a heterogeneous group of non-carbohydrate plant cell wall compounds (chitins, lignins and waxes) and carbohydrate polymers that are neither digested nor absorbed by the human intestine (non-starch polysaccharides, resistant oligosaccharides and resistant starch) [1]. Its recommended dietary intake by the European Food Safety Authority (EFSA) is 25 g per day and it is naturally present in whole foods, fruits, vegetables, tuberous roots and legumes [2].

DF is considered an essential component of a healthy diet and has been associated with several health benefits. A high dietary fiber intake has been related to a significant reduction in mortality from cardiovascular events [3,4], a lower incidence of diverticular disease [5] and a reduced risk of malignancies such as colorectal cancer [6]. However, the main functional property of DF is its prebiotic activity due to fermentation of intestinal microbiota of the colon. Saccharolytic bacteria are capable of producing short-chain fatty acids (SCFAs) from DF, such as butyric, propionic and acetic acids [7], which are associated with a wide range of health properties. Different authors have evidenced the anticancer [8], anti-inflammatory [9], immunoregulatory [10], anti-obesity [11] and hepatoprotective [12] activity of these compounds.

According to their physical characteristics, there are two types of dietary fiber, insoluble dietary fiber (IDF) and soluble dietary fiber (SDF). SDF presents better bioactive properties than IDF due to its fermentability and viscosity [13]. In addition, it has the most desirable health effects, contributing to reducing the risk of several systemic and chronic diseases such as type 2 diabetes, obesity, hypercholesterolemia or colorectal cancer [14]. It has been shown that an intake of about 6 g/day of SDF can be associated with several health benefits, such as reduction of serum LDL-cholesterol and estimated risk of coronary heart disease [15]. For this reason, DF composition and the ratio of soluble dietary fiber (SDF)/insoluble dietary fiber (IDF) are important for nutrition, with a recommended SDF/IDF proportion of 1:2 [16].

On the other hand, fiber from plant by-products is mainly composed of IDF [17]. Therefore, the modification and degradation of IDF is considered of special interest to improve its activities and functional properties. In order to transform IDF into SDF content of food products, chemical, biological or physical techniques have been developed by several researchers. Ultrasound treatment [18], microfluidization [19], use of supercritical fluids [20], or microwave degradation [21] are techniques in increasing development. However, the most effective and most widely used is enzymatic treatment.

In fact, enzymatic hydrolysis is the most promising technology due to higher product yield and low energy requirements. Although its cost is usually higher than other physical methods due to enzyme purification [22], this could be considered an environmentally friendly process because it does not use solvents or chemical reagents [23]. Enzymatic modification consists of using enzymes capable of cleaving insoluble polysaccharides present in food matrices, decomposing them into small molecules. Enzymes have the advantage that they are non-toxic, easy to inactivate and can be highly effective at relatively low concentrations [24]. Moreover, their activity can be regulated by controlling the temperature and pH of the medium. Enzymatic treatment has been used extensively by several authors with the aim of treating different foods, improving their nutritional characteristics. Cellulases, pectinases or xylanases have currently been used in different by-products of potato [25], tomato [26], purple turnip [27], rice bran [28], ginger [29] or date [30].

On the other hand, enzymatic treatment of fibers provides other benefits. Numerous studies have reported that enzyme-assisted extraction is a useful technique for the mobilization of phenolic compounds from glycosides [31]. The extracted phenolic compounds can improve the stability and shelf life of food products, increase their antioxidant capacity and inhibit the growth of a number of microorganisms. Napolitano et al. [32] demonstrated that the conversion of IDF to SDF from cereal fiber by enzymatic treatment achieved an increase in free phenolic concentration, water-soluble antioxidant activity as well as bioavailability of phenolic compounds.

Enzymatic treatment has also been shown to modify the physicochemical and structural characteristics of dietary fibers. The modification of IDF by SDF confers to the food matrices a higher gelling capacity and viscosity as well as better fermentability. Numerous studies consider enzyme-treated fibers as potential thickener, emulsifier, stabilizer and fat substitute [33,34]. These aspects seem to validate the possibility of the application of enzyme-treated food by-products as an innovative form of revalorization in the food industry.

The objective of the current study is to test the possible application of different commercial enzymes (Pectinex^®^ Ultra SP-L, Viscozyme^®^ L and Celluclast^®^ 1.5 L) on different food by-products obtained during the processing of artichoke, broccoli, apple and carob. This will be carried out by validating the conversion of IDF to SDF and the change in the physicochemical characteristics and antioxidant activity of the extracts after treatment, thereby verifying the potential revalorization of the extracts as a possible food additive.

## 2. Materials and Methods

### 2.1. Materials

Extracts of broccoli (BE) (stems), artichoke (ARE) (external bracts), apple (APE) (by-product generated after the production of apple jam) and carob (CE) (fiber obtained after the production of carob syrup) were supplied by Cricket (Lorca, Spain) and local grocery stores. By-products were oven-dried at 50 °C for 18 h. Then, dried by-products were grounded with a coffee mill and sieved until obtaining a fine flour of 500 μm particle size.

Pectinex^®^ Ultra SP-L (blend of pectinases, hemicellulases and beta-glucanases from *Aspergillus aculeatus*; enzyme activity: ≥3300 EGU/g), Viscozyme^®^ L (blend of beta-glucanases, pectinases, hemicellulases and xylanasee from *Aspergillus aculeatus*; enzyme activity: ≥ 100 FBG/g), and Celluclast^®^1.5 L (cellulase from *Trichoderma reesei*; enzyme activity: ≥ 700 PGNU/g) was purchased from Novozymes (Bagsværd, Denmark).

### 2.2. Enzymatic Treatment

By-product extracts were subjected to enzymatic treatment using three different commercial enzymes. For this purpose, diluted extracts (1:5 *w*/*v*) were incubated with Pectinex^®^ Ultra SP-L (65 °C, pH 5.5), Viscozyme^®^ L (45 °C, pH 4.5) and Celluclast^®^1.5 L (50 °C, pH 6) at a concentration of 0.45% (*v*/*w*) for 24 h in an orbital shaker, attending to the most optimal conditions indicated by the provider. Each treatment was done in triplicate for every extract.

After hydrolysis, the mixture was oven-dried at 60 °C and the dried by-product extracts were grounded with a coffee mill and sieved until obtaining a fine flour of 500 μm particle size. The drying process was carried out until the final moisture content of the extracts was approximately 4%. The enzymatic process yield of the extracts was calculated as follows:

(1) $$Yield\left(\%\right)=\frac{Initial\;weight\;\left(g\right)}{Final\;weight\;\left(g\right)}\times 100$$

### 2.3. Determination of Physicochemical Characteristics

Solubility measurement was performed following the method described by Cano-Chauca et al. [35] with some modifications. For 5 min, 0.5 g of sample was mixed with 50 mL of water. The solution was placed in a tube and centrifuged at 3000× *g* for 5 min. A 12.5 mL aliquot of the supernatant was transferred to previously weighed Petri dishes and immediately oven-dried at 100 °C for 5 h. Each treatment was done in triplicate for every extract. The solubility (%) was calculated by weight difference according to the following formula:

(2) $$Solubility\left(\%\right)=\frac{Dried\;Supernatnt\;weight\;\left(g\right)\times 4}{Sample\;weight\;\left(g\right)}\times 100$$

Water-holding capacity (WHC) was determined following the protocol described by Luo et al. [36]. In a centrifuge tube, 1 g of dry sample was mixed with 20 mL of distilled water for 24 h at room temperature. Samples were centrifuged at 3000 rpm for 20 min, the supernatant was carefully discarded and the pulp residue was collected and weighed. WHC was expressed as grams of water per gram of dry sample.

Fat-adsorption capacity (FAC) was calculated with the method described by Sosulsky and Cadden [37]. For this purpose, 1 g of sample was mixed with sunflower oil for 30 min. Afterwards, the samples were centrifuged at 1600× *g* for 25 min. FAC was calculated as the amount of sunflower oil retained by each sample expressed as g/g.

Color was measured using a Konica Minolta CR 400 chromameter (Minolta Camera Co., Osaka, Japan), standardized using a calibration plate. Lightness (L*), a* chromaticity (red-green) and b* chromaticity (blue-yellow) were measured according to the CIELab system. Measurements were taken in triplicate on untreated and enzymatically treated extracts.

The total color differences (ΔE) of each sample with respect to the control formulation and in relation to day 0 of storage were calculated as follows:

(3) $$∆E=\sqrt{{\left(L_{s}^{*}-L_{c}^{*}\right)}^{2}+{\left(a_{s}^{*}-a_{c}^{*}\right)}^{2}+{\left(b_{s}^{*}-b_{c}^{*}\right)}^{2}}$$

### 2.4. Dietary Fiber Quantification

Total dietary fiber (TDF), soluble dietary fiber (SDF) and insoluble dietary fiber (IDF) were determined according to the 985.29:1985 method of the Association of Official Analytical Chemists (AOAC), described by Prosky et al. [38].

For the determination, 0.5 g of each sample extract was weighed in triplicate and diluted with 25 mL of phosphate buffer (50 mM, pH 6). To facilitate starch hydrolysis, samples were incubated with 50 µL of α-amylase enzyme from *Bacillus licheniformis* (500–1500 units/mg protein) (Sigma-Aldrich, Saint Louis, MO, USA) for 30 min at 95 °C in a shaking water bath. Subsequently, the samples were subjected to a second digestion (pH 7.5, 60 °C, 30 min) by adding 100 µL of 0.5 mg/mL protease from *Bacillus licheniformis* (7–15 units/mg solid) (Sigma-Aldrich, Saint Louis, MO, USA). Finally, 150 µL of amyloglucosidase from *Aspergillus niger* (≥260 units/mL) (Sigma-Aldrich, Saint Louis, MO, USA) was added to the mixture, and incubated for 30 min at 60 °C.

After digestion, filtration of the dietary fiber was carried out using a Fibertec System E 1023 (Foss, Högänas, Sweden). An initial filtration was performed to isolate the IDF. The filtrate from the previous step was precipitated after the addition of 140 mL of 95% ethanol at 60 °C and allowed to stand for one hour. The filtration procedure was then repeated and the SDF residue was collected. Once the residues were obtained, the protein and ash content of the residues were analyzed through standard methods of AOAC [39] (955.04:1995 and 923.03:1995, respectively). TDF content was calculated as the sum of IDF and SDF. The Dietary Fiber Conversion rate (DFC) from IDF to SDF was calculated as follows:

(4) $$DFC\;\left(\%\right)=\frac{{SDF}_{AfterTreatment}-{\;SDF}_{Before\;Treatment}}{{IDF}_{BeforeTreatment}}\times 100$$

### 2.5. Total Phenolic Content (TPC) and Antioxidant Capacity

Before the analysis, the different formulations of artichoke, broccoli, carob and apple extracts were homogenized in a methanol/water solution (80%, *v*/*v*). The mixture was incubated for 24 h at 4 °C and centrifuged (4500 rpm, 25 min). Supernatants were filtered (0.45 µm) and kept at −18 °C until analysis.

The total phenolic content (TPC) was determined following the method described by Singleton et al. [40] using Folin–Ciocalteau reagent and gallic acid as the standard. The analysis was carried out in triplicate and the TPC was expressed as mg of gallic acid equivalents (GAEs) per g of sample.

The ferric-reducing antioxidant power (FRAP) assay was determined following the protocol described by Benzie and Strain [41]. The FRAP reagent was prepared with a solution of 300 mmol/L acetate buffer, pH = 3.6, 20 mmol/L FeCl_3_·6 H_2_O and 10 mmol/L TPTZ (2,4,6-tripyridyl-s-triazine) in 40 mmol/L HCl (10:1:1, *v*/*v*/*v*). A 100 µL amount of sample was mixed with 1 mL of FRAP reagent in plastic cuvettes and absorbance was measured at 593 nm after 4 min of incubation. The same procedure was carried out with a standard solution of Trolox. Analyses were carried out in triplicate and the antioxidant capacity was expressed as µM Trolox equivalents (TEs) per g of sample.

Antioxidant activity related to chelating capacity was measured using the 2,2-diphenyl-1-picrylhydrazyl (DPPH) free radical scavenging method described by Brand-Williams et al. [42]. A 100 µL amount of sample was mixed with 3.9 mL DPPH reagent in plastic cuvettes and absorbance was measured at 515 nm after 30 min of incubation in the dark. The same procedure was carried out with a standard solution of Trolox. Analyses were carried out in triplicate and antioxidant activity related to chelating capacity was expressed as µM Trolox equivalents (TEs) per g of sample.

The radical cation scavenging capacity against 2,2-azinobis (3-ethylbenzothiazolin)-6-sulphonic acid (ABTS) radical scavenging was carried out following the method described by Re et al. [43]. The ABTS reagent (7 mM) was prepared in 2.45 mM potassium persulphate (1:1, *v*/*v*), pH = 7.4, and adjusted to an absorbance of 0.7000 at 734 nm. A 1 mL amount of ABTS reagent was mixed with 100 µL of sample and absorbance was measured at 734 nm after 2 min of incubation. Analyses were carried out in triplicate and antioxidant activity related to radical scavenging capacity was expressed as µM Trolox equivalents (TEs) per g of sample.

### 2.6. Statistical Analysis

Differences between extracts were determined by one-way analysis of variance (ANOVA). A value of *p* < 0.05 was considered statistically significant, using the Tukey post-hoc test. Pearson’s correlation was applied to test correlations between all studied markers. The results were expressed as mean ± standard deviation (SD). All statistical analyses were performed using IBM SPSS Statistics 28.0 software (IBM Corporation, Armonk, NY, USA).

## 3. Results and Discussion

### 3.1. Dietary Fiber Analysis

**Figure 1:**
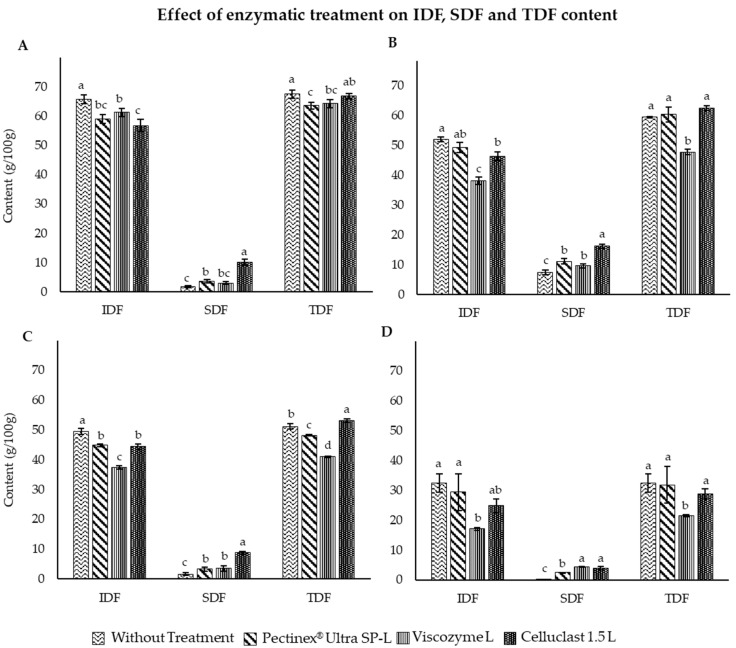
Effect of enzymatic treatment with different enzymes on IDF, SDF and TDF content of by-product extracts of CE (**A**), APE (**B**), ARE (**C**) and BE (**D**). ^a–d^: Different letters for each parameter indicate significant differences between enzyme treatments (*p* < 0.05).

The IDF, SDF and TDF content of carob, artichoke, apple and broccoli extracts, as well as after application of enzymatic treatment using Pectinex^®^ Ultra SP-L, Viscozyme^®^ L and Celluclast 1.5 L, all purchased from Novozymes (Bagsværd, Denmark), are represented in Figure 1.

The results obtained show a high fiber content for the four extracts, with TDF representing 67.5, 59.5, 51.1 and 32.5% for carob, apple, artichoke and broccoli, respectively. SDF and IDF results were in accordance with the established literature for carob (65.69% IDF and 1.81% SDF) [44]. The enzymatic treatment applied to this extract significantly increased the SDF values and reduced the IDF values (Figure 1A). Particularly, the treatment with Celluclast 1.5 L achieved the best results, being able to convert up to 12.59% (Table 1) of the insoluble to soluble fiber content of carob by-product extract. Similar results were obtained for APE, in which the enzymatic treatment significantly reduced the levels of IDF, highlighting the results obtained with Viscozyme^®^ L, where a more pronounced decrease was produced. However, the most remarkable increase for soluble fiber content were obtained with the enzyme Celluclast 1.5 L, resulting in a DFC value of 17.01%. Celluclast 1.5 L also obtained the best results after the enzymatic treatment of artichoke, being able to increase SDF content from 1.68 to 8.78% (Figure 1C). However, Viscozyme^®^ L treatment was able to reduce the IDF content to a greater extent. Finally, the treatment also exerted a positive effect on broccoli extract. In this case, Viscozyme^®^ L and Celluclast^®^ 1.5 L enzymes exerted a similar effect after treatment, achieving an IDF to SDF turnover of 13.39 and 11.82%, respectively.

Overall, Celluclast^®^ 1.5 L was the enzyme that caused the greatest increase in SDF in carob, apple and artichoke extracts, with Viscozyme^®^ L being the most effective after the enzymatic treatment of broccoli.

These results may be due to the fact that Celluclast^®^ 1.5 L is an enzyme that catalyzes the hydrolysis of the β-1,4-glycosidic bond of cellulose and hemicellulose, developing cellulo-, xylan- and mannolytic activities [45], giving place to smaller monosaccharides and polysaccharides (cellobiose, cello-oligosaccharides or glucose). Cellulose and hemicellulose are the main components of IDF in artichoke [46], apple [45] and carob [47], so an increase in SDF and a decrease in IDF is probably the result of enzymatic cleavage of cellulose and hemicellulose by this enzyme. On the other hand, broccoli stems present a high content of cellulose, hemicellulose and pectins [48], so both Celluclast^®^ 1.5 L and Viscozyme^®^ L (enzyme cocktail containing xylanase, arabanase, β-glucanase, hemicellulose and cellulase) are very effective in this extract. Finally, Pectinex^®^ Ultra SP-L is a polygalacturonase, an enzyme only able to hydrolyze pectins (soluble fiber), and unable to break cellulose bonds. For this reason, this commercial enzyme is more specific than Celluclast^®^ 1.5 L and Viscozyme^®^ L, showing the lowest results in the conversion of IDF to SDF.

Several authors have studied the beneficial effect of enzymatic treatment on extracts from by-products. Sabater et al. [49] examined the effect of Celluclast^®^ 1.5 L on external bracts, leaves and stems of artichoke, demonstrating a high capacity for fiber hydrolysis and improving the extraction of pectins. Fissore et al. [50] reported similar results in enzymatically treated artichoke by-products, increasing the content of inulin, the main compound present in the SDF of artichoke. In addition, the effect of cellulase and Viscozyme^®^ L has also been tested in extracts of leaves, stems and inflorescences of broccoli, with both being enzymes able to increase the content of soluble fibers such as pectins.

The application of pectinases as enzymatic treatment in apple by-products is the most widely used [24,51,52]. Our results, as well as recent studies conclude that cellulases can induce a higher hydrolytic effect of apple fiber, enhancing the release of oligosaccharides [53] and soluble fibers [53,54]. Jagelaviciute et al. [52] found that hydrolysis with Celluclast^®^ 1.5 L increased content of SDF and SDF/IDF ratio in apple pomace. However, enzymatic treatment with Viscozyme^®^ L and Pectinex^®^ Ultra Tropical resulted in decreased SDF/IDF ratio.

Moreover, Table 2 summarizes the yield of the extracts obtained after enzymatic treatment. The yield was calculated according to formula (2) described in Section 2.2 of the “Materials and Methods” section.

Despite being the enzyme with the lowest soluble fiber-conversion potential, the best yield results for CE, ARE and BE were achieved by the Pectinex^®^ Ultra SP-L treatment (84.13–87.13%). The high yield of pectinase application compared to other enzymatic treatments has been described by other authors [51,52]. The degradation of pectin in the primary matrix of the cell wall and in the middle lamella is necessary to break the water-binding capacity of pectin, increasing the hydration properties of the samples due to the hydroxyl groups that allow water associations through hydrogen bonds [51], and therefore increasing the yield obtained.

On the other hand, similar results were obtained for the extracts treated with Viscozyme^®^ L and Celluclast^®^ 1.5 L. Both enzymatic treatments developed an acceptable yield for all extracts, enabling their potential use for dietary fiber modification.

### 3.2. Physicochemical Characteristics

Solubility properties constitute an important parameter for functional ingredients, as they are related to the structure of polysaccharides; they can be established as regular (insoluble) or irregular (soluble) in the skeleton or as side chains [55]. The solubility values of the different extracts after enzymatic treatment are shown in Table 3.

Solubility values obtained showed a clear relationship between its increase and the enzymatic treatment. The untreated extracts presented the lowest solubility results, showing differences between extracts, although BE showed the highest values and CE the lowest values. The increase in solubility for each extract relied on the enzyme used during treatment. Viscozyme^®^ L was the most effective for CE and BE, achieving an increase in solubility of 13.76 and 24.28%, respectively. On the other hand, the treatment with Pectinex^®^ Ultra SP-L was the best performer for APE, increasing by 13.13%. Finally, Celluclast^®^ 1.5 L was the most effective for ARE, increasing solubility values from 24.88% to 44.49%.

Similar results were obtained for water-holding capacity (WHC) and fat-adsorption capacity (FAC). A general increase in fluid retention was observed after the application of enzymatic treatment in CE, APE, ARE and BE. In particular, a greater increase was found in the extracts treated with Pectinex^®^ Ultra SP-L, reaching increases in WHC of 163, 118, 130 and 109% for CE, APE, ARE and BE, respectively. The possible reason for the improvement in solubility and liquid retention may be certain changes in the microstructure increasing the surface area of the modified dietary fiber, exposing in turn more water-binding sites, which favored the absorption and penetration of water molecules [33,56]. Other authors have demonstrated this improvement in water solubility after enzymatic treatment. Similar results for extracts from broccoli by-products were obtained by Rivas et al. [57], reaching water solubility values of 53.7% after treatment with Viscozyme^®^ L, as well as an increase in WHC and FAC in leaves, stems and inflorescences of broccoli. In addition, the effect on solubility that pectinases can induce in apple extracts has also been demonstrated [24], with some authors reporting the highest solubility index (43.80 ± 2.10%) obtained using Pectinex^®^ Ultra SP-L [52]. Pectin can increase the hydration properties of the samples due to its hydroxyl groups that allow water associations through hydrogen bonds, which could be the reason it is the enzyme that gives the highest results for WHC and FAC.

Moreover, it has been shown that the effect of cellulase in carob juice can increase the water-retention capacity and, consequently, a reduction in the viscosity of the juice and facilitating filtration [58]. On the other hand, it has been possible to improve the water-holding capacity of extracts from other food matrices applying different enzymes, such as potato pulp using Viscozyme^®^ L [25], purple turnip using cellulases [27] or corn bran using xylanases and cellulases [59]. As expected, these improvements in solubility correspond with the results obtained for SDF content, reaffirming Celluclast^®^ 1.5 L as the most effective enzyme for APE and ARE, and Viscozyme^®^ L as the most appropriate for BE treatment.

It has been shown that dietary fiber with high solubility and WHC could be used as a functional ingredient to modify the viscosity and texture of some foods (bakery products, dairy products, jams, meats, soups), as well as to stabilize fat in emulsion-based products [60], and to improve shelf life [55]. Some studies also evidenced that foods with high water-holding capacity and solubility prevent and treat intestinal disorders by increasing fecal bulk and reducing gastrointestinal transit time [57], and may have a positive impact on reducing the risk of chronic disorders, coronary heart disease, diabetes, obesity and some forms of cancer [55].

On the other hand, Table 4 summarizes the color (CIElab) measurements of the untreated and enzymatically treated extracts.

Overall, significant differences (*p* < 0.05) were observed between the results of the L*, a* and b* coordinates between the extracts before and after enzymatic treatment, showing that this has an impact on the color. A loss of reddish color (a*) was found after treatment with all enzymes in APE, ARE and BE, while only Pectinex^®^ Ultra SP-L changed the color in CE. In addition, changes in the blue-yellow chromaticity (b*) and Lightness (L*) were also observed, being reduced during all treatments of APE, ARE and BE. A parameter that was taken into account when evaluating color modifications during treatment is the total color difference (∆E) relative to the original sample. Several studies have mentioned that ∆E values greater than 3 units correspond to changes perceptible to the human eye [61]. After analysis, a significant change greater than 3 was observed for all treatments in CE, APE, ARE and BE, denoting a clear relationship between the enzymatic treatment and a visually appreciable color change. In addition, a greater change was seen in the APE and BE extracts, reaching values above 10 after the use of the three different enzymes. On the other hand, CE was the extract that underwent the least color change, especially after treatment with Viscozyme^®^ L.

Several studies have examined the impact that enzymatic treatment can have on the color of extracts. Pectinases and cellulases have been used to change the color of saffron tepals [62] or black tea [63] extracts, improving their acceptability. Konopacka et al. [64] observed changes in L*, a* and b* after enzymatic treatment of pumpkin and carrot, increasing these values after application of commercial pectinases. The reduction of L*, a * and b* values obtained after treatment may be due to a browning effect of the extracts. The application of temperatures above 50 °C during processing may accelerate a chemical reaction of the polyphenol oxidase enzyme, catalyzing the production of melanins and benzoquinone from polyphenols present in the extracts [65].

### 3.3. Antioxidant Activity and Total Phenolic Content (TPC) Determination

The antioxidant capacity of the extracts (FRAP, ABTS and DPPH) before and after enzymatic treatment as well as the total phenolic content (TPC) are summarized in Table 5. Treatment with different enzymes caused significant changes (*p* < 0.05) in antioxidant activity. A significant increase in DPPH was observed for CE, after the use of Pectinex^®^ Ultra SP-L, Viscozyme^®^ L and Celluclast^®^ 1.5 L, while positive trends were observed in FRAP and ABTS assays. Concerning ARE, Pectinex^®^ Ultra SP-L and Celluclast^®^ 1.5 L were able to significantly increase the ABTS values with respect to the untreated extract. The results for APE were also positive, as the Viscozyme^®^ L treatment significantly increased the antioxidant capacity in the FRAP, ABTS and DPPH assays, increasing by 149, 156 and 171%, respectively. Finally, significant changes were also observed in BE, with Pectinex^®^ Ultra SP-L and Viscozyme^®^ L treatments being beneficial in ABTS and DPPH tests.

Similar results have been found in the literature, Napolitano et al. [32] showed that the conversion of IDF to SDF from barley spent grain and durum wheat fiber produced an increase in antioxidant activity measured by ABTS assay after enzymatic treatment. Another study performed on black currant extracts showed an increase in the inhibition of human LDL Oxidation after treatment with commercial enzymes (Pectinex BE, Macer8 FJ and Novozyme) [66]. Mrabet et al. [30] also demonstrated that treatment with Viscozyme^®^ L produced an increase in the DPPH assay in date fruit. On the other hand, the results for total phenolic content were more diverse. No significant differences were observed after CE enzyme treatment; however, an increase in TPC was observed in Celluclast^®^ 1.5 L-treated ARE and a decrease after Viscozyme^®^ L treatment. The results with ARE showed a clear relationship between enzyme treatment and higher polyphenol content, as both Viscozyme^®^ L and Celluclast^®^ 1.5 L treatments increased TPC levels. Finally, the results showed an increase after treatment with Pectinex^®^ Ultra SP-L and Viscozyme^®^ L.

These results show that there is a relationship between the application of different enzymes and a higher amount of polyphenols depending on the extract used. This may be because phenols that were trapped or structurally bound to the fiber could be partially released after digestion and are observed in a higher quantity [67].

Similar results were found in the research of Jagelaviciute et al. [52] where an increase in TPC of apple pomace was found after treatment with Viscozyme^®^ L, Pectinex^®^ Ultra Tropical and Celluclast^®^ 1.5 L. In fact, these results also agree with those obtained by Landbo and Meyer [66] where the use of different pectinases increased the total phenolic content of black currant. On the other hand, it has also been shown that the conversion of IDF to SDF from coffee silver skin by enzymatic treatment can also lead to the release of hydroxycinnamic acids, mainly ferulic acid, which are bound to polysaccharide chains [32].

### 3.4. Pearson Correlations

A Pearson analysis was performed to determine the correlation of the different parameters studied; the results are presented in Table 6.

The results obtained showed significant differences (*p* < 0.05) between the different parameters studied. A relationship was observed between the different color parameters (a*, b*, L* and ∆E) and soluble fiber content, suggesting that enzymatic treatment influences the color of the extracts, making them darker. As mentioned above, a possible hypothesis for this phenomenon is enzymatic browning due to temperatures above 50 °C during the enzymatic treatment process, thereby accelerating a chemical reaction of the polyphenol oxidase enzyme and catalyzing the production of melanins from the polyphenols present in the extracts [65].

Another aspect to highlight is the significant relationship between SDF content and solubility; this may be due to the fact that certain changes in the structure increased the surface area of the modified dietary fiber, in turn exposing more water-binding sites, which favored the absorption of water molecules [56]. However, the relationship between SDF and WHC/FAC was not significant, indicating that there are more factors to consider between these parameters.

## 4. Conclusions

Enzymatic treatment of food by-products with different commercial enzymes achieved considerable nutritional and physicochemical benefits. Increases in SDF levels were demonstrated for all by-products, with the treatment being especially positive with Viscozyme^®^ L for BE and Cellucalst^®^ 1.5 L for CE, ARE and APE. Moreover, this increase in the SDF content was related to an improvement in the solubility, WHC and FAC of the extracts, which showed an increase after almost all possible enzymatic treatments. Additionally, treatment with the different enzymes had an impact on the antioxidant capacity of all the extracts, conferring them health-promoting benefits. On the other hand, the processes maintained a high yield after enzyme application, indicating a good potential application of this technology on a larger scale. These results evidence potential benefits of different extracts after the application of an enzymatic treatment, which could represent a key opportunity for the food industry and an attractive revaluation for food by-products.

## Figures and Tables

**Table 1:** Dietary Fiber Conversion rate (DFC) of IDF to SDF (%).

Treatment	Extracts			
	CE	APE	ARE	BE
Without Treatment	0.00 ± 0.00 ^c^	0.00 ± 0.00 ^c^	0.00 ± 0.00 ^c^	0.00 ± 0.00 ^c^
Pectinex^®^ Ultra SP-L	2.73 ± 0.49 ^b^	7.22 ± 1.54 ^b^	3.27 ± 1.00 ^b^	7.37 ± 0.57 ^b^
Viscozyme^®^ L	1.85 ± 0.32 ^bc^	4.34 ± 2.08 ^b^	3.82 ± 0.48 ^b^	13.39 ± 2.07 ^a^
Celluclast^®^ 1.5 L	12.59 ± 1.36 ^a^	17.01 ± 1.55 ^a^	14.38 ± 1.76 ^a^	11.82 ± 0.91 ^a^

^a–c^: Different letters within in the same column indicate significant differences between enzymatic treatments (*p* < 0.05). APE: apple extract; ARE: artichoke extract; BE: broccoli extract; CE: carob extract.

**Table 2:** Yield (%) after enzymatic treatment of the by-product extracts.

Treatment	Extracts			
	CE	APE	ARE	BE
Pectinex^®^ Ultra SP-L	87.13 ± 0.83 ^a^	81.44 ± 0.39 ^a^	86.97 ± 0.67 ^a^	84.13 ± 0.32 ^a^
Viscozyme^®^ L	78.42 ± 0.67 ^b^	83.72 ± 1.12 ^a^	81.55 ± 0.48 ^b^	78.32 ± 0.21 ^b^
Celluclast^®^ 1.5 L	86.70 ± 0.87 ^a^	79.15 ± 1.23 ^a^	80.24 ± 0.57 ^b^	82.17± 0.67 ^a^

^a,b^: Different letters in the same column indicate significant differences between enzymatic treatments (*p* < 0.05). APE: apple extract; ARE: artichoke extract; BE: broccoli extract; CE: carob extract.

**Table 3:** Solubility, WHC and FAC measurements after enzymatic treatment.

Treatment	Extracts			
	CE	APE	ARE	BE
Solubility (%)				
Without Treatment	17.41 ± 2.91 ^b^	29.25 ± 0.34 ^c^	24.88 ± 0.29 ^c^	38.69 ± 0.51 ^c^
Pectinex^®^ Ultra SP-L	21.55 ± 3.51 ^b^	42.38 ± 0.62 ^a^	36.21 ± 0.41 ^b^	29.20 ± 0.67 ^d^
Viscozyme^®^ L	31.17 ± 0.88 ^a^	36.83 ± 0.27 ^b^	37.49 ± 0.89 ^b^	62.97 ± 0.62 ^a^
Celluclast^®^ 1.5 L	22.04 ± 4.03 ^b^	42.37 ± 0.26 ^a^	44.49 ± 0.21 ^a^	58.04 ± 0.08 ^b^
WHC (g/g)				
Without Treatment	2.32 ± 0.16 ^b^	1.45 ± 0.06 ^c^	2.89 ± 0.07 ^c^	1.91 ± 0.09 ^a^
Pectinex^®^ Ultra SP-L	3.79 ± 0.63 ^ab^	1.72 ± 0.04 ^bc^	3.77 ± 0.04 ^a^	2.11 ± 0.00 ^a^
Viscozyme^®^ L	2.63 ± 0.03 ^ab^	1.84 ± 0.12 ^b^	3.33 ± 0.05 ^b^	1.79 ± 0.18 ^a^
Celluclast^®^ 1.5 L	3.11 ± 0.30 ^b^	2.37 ± 0.07 ^a^	3.63 ± 0.05 ^a^	2.13 ± 0.14 ^a^
PAC (g/g)				
Without Treatment	1.03 ± 0.02 ^c^	0.77 ± 0.02 ^a^	1.69 ± 0.00 ^c^	0.56 ± 0.01 ^c^
Pectinex^®^ Ultra SP-L	3.64 ± 0.13 ^a^	1.08 ± 0.03 ^a^	2.69 ± 0.03 ^ab^	0.97 ± 0.02 ^b^
Viscozyme^®^ L	1.40 ± 0.06 ^ab^	0.71 ± 0.20 ^a^	2.38 ± 0.15 ^b^	0.92 ± 0.03 ^bc^
Celluclast^®^ 1.5 L	1.32 ± 0.02 ^b^	1.13 ± 0.06 ^a^	2.90 ± 0.18 ^a^	1.07 ± 0.03 ^a^

^a–d^: Different letters in the same column indicate significant differences between enzymatic treatments (*p* < 0.05). APE: apple extract; ARE: artichoke extract; BE: broccoli extract; CE: carob extract.

**Table 4:** Color evolution (CIELab) of untreated and treated extracts.

Treatment	CE	APE	ARE	BE
L*				
Without Treatment	44.20 ± 1.93 ^a^	50.83 ± 2.17 ^a^	54.39 ± 0.42 ^a^	54.8 ± 2.02 ^a^
Pectinex^®^ Ultra SP-L	46.94 ± 1.21 ^a^	39.77 ± 0.48 ^c^	49.41 ± 0.13 ^b^	46.56 ± 0.11 ^b^
Viscozyme^®^ L	41.19 ± 0.01 ^b^	36.76 ± 0.76 ^c^	47.73 ± 0.46 ^ca^	43.64 ± 0.72 ^c^
Celluclast^®^ 1.5 L	39.80 ± 0.31 ^b^	44.30 ± 2.10 ^b^	49.20 ± 0.09 ^b^	48.97 ± 0.29 ^b^
a*				
Without Treatment	11.17 ± 0.12 ^a^	12.19 ± 0.32 ^a^	8.74 ± 0.07 ^a^	4.94 ± 0.27 ^a^
Pectinex^®^ Ultra SP-L	4.62 ± 0.04 ^c^	9.12 ± 0.02 ^b^	4.84 ± 0.03 ^c^	3.64 ± 0.07 ^b^
Viscozyme^®^ L	11.07 ± 0.03 ^a^	8.51 ± 0.03 ^c^	6.50 ± 0.03 ^b^	3.74 ± 0.26 ^b^
Celluclast^®^ 1.5 L	10.50 ± 0.08 ^a^	8.74 ± 0.28 ^bc^	4.68 ± 0.14 ^c^	3.83 ± 0.04 ^b^
b*				
Without Treatment	19.03 ± 0.39 ^a^	28.47 ± 0.31 ^a^	26.27 ± 0.45 ^a^	27.62 ± 0.26 ^a^
Pectinex^®^ Ultra SP-L	19.35 ± 0.20 ^a^	19.51 ± 0.23 ^b^	21.51 ± 0.07 ^ab^	19.67 ± 0.11 ^b^
Viscozyme^®^ L	18.24 ± 0.01 ^b^	16.83 ± 1.29 ^c^	21.93 ± 0.08 ^b^	17.82 ± 0.75 ^c^
Celluclast^®^ 1.5 L	16.46 ± 0.10 ^c^	17.90 ± 0.37 ^bc^	21.29 ± 0.14 ^c^	20.51 ± 0.19 ^b^
∆E				
Without Treatment	0.00 ± 0.00 ^c^	0.00 ± 0.00 ^c^	0.00 ± 0.00 ^b^	0.00 ± 0.00 ^c^
Pectinex^®^ Ultra SP-L	7.13 ± 0.30 ^a^	14.61 ± 2.08 ^ab^	7.92 ± 0.28 ^a^	11.59 ± 1.57 ^ab^
Viscozyme^®^ L	3.20 ± 1.95 ^bc^	18.79 ± 0.45 ^a^	8.27 ± 0.09 ^a^	14.95 ± 0.79 ^a^
Celluclast^®^ 1.5 L	5.30 ± 1.58 ^ab^	10.08 ± 2.87 ^b^	8.27 ± 0.33 ^a^	9.33 ± 1.21 ^bc^

^a–c^: Different letters in the same column indicate significant differences between enzymatic treatments (*p* < 0.05). APE: apple extract; ARE: artichoke extract; BE: broccoli extract; CE: carob extract.

**Table 5:** Antioxidant activity (µmol TE/g) and Total Phenolic Content (TPC) (mg GAE/g) after enzymatic treatment of the by-product extracts.

Assay	Treatment	CE	APE	ARE	BE
FRAP	Without Treatment	140.81 ± 37.74 ^a^	94.70 ± 11.03 ^a^	123.22 ± 2.45 ^b^	74.10 ± 2.17 ^a^
	Pectinex^®^ Ultra SP-L	163.32 ± 0.54 ^a^	98.94 ± 16.92 ^a^	130.47 ± 4.32 ^b^	84.67 ± 2.98 ^a^
	Viscozyme^®^ L	179.07 ± 4.38 ^a^	63.69 ± 0.27 ^b^	182.12 ± 4.90 ^a^	85.56 ± 7.10 ^a^
	Celluclast^®^ 1.5 L	181.60 ± 18.6 ^a^	113.79 ± 4.09 ^a^	130.61 ± 2.73 ^b^	65.22 ± 7.37 ^a^
ABTS	Without Treatment	124.40 ± 20.49 ^b^	58.22 ± 0.39 ^ab^	80.92 ± 12.35 ^b^	55.83 ± 1.19 ^b^
	Pectinex^®^ Ultra SP-L	171.90 ± 3.97 ^ab^	82.47 ± 0.00 ^ab^	83.58 ± 6.00 ^b^	68.51 ± 2.60 ^a^
	Viscozyme^®^ L	179.63 ± 14.41 ^ab^	41.02 ± 4.39 ^b^	126.10 ± 1.20 ^a^	76.66 ± 0.40 ^a^
	Celluclast^®^ 1.5 L	198.32 ± 18.00 ^a^	85.55 ± 11.47 ^a^	85.86 ± 4.34 ^b^	54.01 ± 2.79 ^b^
DPPH	Without Treatment	199.00 ± 44.07 ^a^	129.75 ± 31.41 ^a^	126.85 ± 19.08 ^b^	106.06 ± 25.31 ^ab^
	Pectinex^®^ Ultra SP-L	269.93 ± 31.71 ^a^	129.33 ± 22.31 ^a^	129.20 ± 31.41 ^b^	137.90 ± 3.71 ^a^
	Viscozyme^®^ L	276.81 ± 0.00 ^a^	68.56 ± 3.18 ^a^	217.02 ± 25.47 ^a^	143.06 ± 3.91 ^a^
	Celluclast^®^ 1.5 L	280.93 ± 28.73 ^a^	137.21 ± 22.12 ^a^	174.96 ± 3.20 ^ab^	69.67 ± 7.97 ^b^
TPC	Without Treatment	59.22 ± 24.82 ^a^	74.20 ± 9.32 ^ab^	84.78 ± 0.74 ^b^	63.72 ± 0.74 ^bc^
	Pectinex^®^ Ultra SP-L	71.11 ± 3.75 ^a^	81.44 ± 0.39 ^ab^	85.85 ± 1.72 ^ab^	74.45 ± 2.62 ^ab^
	Viscozyme^®^ L	47.57 ± 0.99 ^a^	53.48 ± 0.50 ^b^	127.91 ± 0.00 ^a^	88.75 ± 5.48 ^a^
	Celluclast^®^ 1.5 L	55.32 ± 1.00 ^a^	83.36 ± 1.97 ^a^	109.2 ± 23.71 ^ab^	51.01 ± 3.49 ^c^

^a–c^: Different letters in the same column indicate significant differences between enzymatic treatments (*p* < 0.05). APE: apple extract; ARE: artichoke extract; BE: broccoli extract; CE: carob extract.

**Table 6:** Pearson correlations between different measured parameters.

	SDF	DFC	FRAP	TPC	DPPH	ABTS	a*	b*	L*	∆E	WHC	FAC	Solubility
SDF		0.729 **	−0.188	0.067	−0.112	−0.105	0.208	−0.447 **	−0.528 **	0.524 **	−0.216	−0.186	0.342 *
DFC	0.729 **		−0.152	0.257	−0.034	−0.029	−0.299 *	−0.533 **	−0.321 *	0.531 **	0.006	−0.028	0.627 **
FRAP	−0.188	−0.152		0.26	0.840 **	0.859 **	0.318	−0.213	−0.154	−0.33	0.663 **	0.574 **	−0.518 **
TPC	0.067	0.257	0.26		0.044	−0.09	−0.189	0.157	0.222	0.044	0.291	0.294	0.29
DPPH	−0.112	−0.034	0.840 **	0.044		0.944 **	0.276	−0.322	−0.235	−0.282	0.517 **	0.459 **	−0.532 **
ABTS	−0.105	−0.029	0.859 **	−0.09	0.944 **		0.311	−0.415 *	−0.322	−0.26	0.531 **	0.421 *	−0.541 **
a*	0.208	−0.299 *	0.318	−0.189	0.276	0.311		0.025	−0.335 *	−0.380 **	−0.242	−0.329	−0.526 **
b*	−0.447 **	−0.533 **	−0.213	0.157	−0.322	−0.415 *	0.025		0.843 **	−0.623 **	−0.142	−0.059	−0.106
L*	−0.528 **	−0.321 *	−0.154	0.222	−0.235	−0.322	−0.335 *	0.843 **		−0.592 **	0.134	0.176	−0.001
∆E	0.524 **	0.532 **	−0.33	0.044	−0.282	−0.26	−0.380 **	−0.623 **	−0.592 **		−0.169	−0.059	0.531 **
WHC	−0.216	0.006	0.663 **	0.291	0.517 **	0.531 **	−0.242	−0.142	0.134	−0.169		0.900 **	−0.297
FAC	−0.186	−0.028	0.574 **	0.294	0.459 **	0.421 *	−0.329	−0.059	0.176	−0.059	0.900 **		−0.208
Solubility	0.342 *	0.627 **	−0.518 **	0.290	−0.532 **	−0.541 **	−0.526 **	−0.106	−0.001	0.532 **	−0.297	−0.208	

Significance levels: *: *p* < 0.05; **: *p* < 0.01.

## Data Availability

The original contributions presented in the study are included in the article, further inquiries can be directed to the corresponding author.
